# Prevalence and Sociodemographic Correlates of Smoking among Schoolchildren in Albania

**DOI:** 10.3390/ijerph21091145

**Published:** 2024-08-29

**Authors:** Rudina Çumashi, Iris Mone, Genc Burazeri, Lulzim Çela, Enkeleint A. Mechili, Gentiana Qirjako

**Affiliations:** 1 Institute of Public Health, Rr. “Aleksander Moisiu”, No. 80, P.O. Box 1005 Tirana, Albania; rudicumashi@gmail.com; 2 Department of Public Health, Faculty of Medicine, University of Medicine, Rr. “Dibres”, No. 371, P.O. Box 1005 Tirana, Albania; genc.burazeri@maastrichtuniversity.nl; 3 Department of Biomedical and Experimental Sciences, Faculty of Medicine, University of Medicine, Rr. “Dibres”, No. 371, P.O. Box 1005 Tirana, Albania; iris_mone@yahoo.com; 4 Department of International Health, CAPHRI (Care and Public Health Research Institute), Maastricht University, 6200 MD Maastricht, The Netherlands; 5 University Hospital Center “Mother Teresa”, Rr. “Dibres”, No. 370, P.O. Box 1005 Tirana, Albania; lulzim_cela@yahoo.com; 6 Department of Healthcare, Faculty of Health, University of Vlora, P.O. Box 9401 Vlora, Albania; mechili@univlora.edu.al; 7 School of Medicine, University of Crete, P.O. Box 71003 Crete, Greece

**Keywords:** Albania, children, e-cigarettes, health behaviour in school-aged children (HBSC), schoolchildren, smoking, tobacco

## Abstract

Smoking among children remains a critical public health issue, with millions of minors engaging in tobacco use, leading to addiction and long-term health consequences. Our objective was to assess the prevalence and sociodemographic distribution of smoking habits among Albanian children. A cross-sectional study was conducted in Albania in 2022, including a nationwide representative sample of 5454 schoolchildren aged 11, 13, and 15 years (N = 5454; ≈52% girls; response rate: 96%). Data on smoking habits were gathered, along with sociodemographic factors of schoolchildren. Binary logistic regression was used to assess the associations of smoking variables with sociodemographic factors. The prevalence of smoking variables was 12% for lifetime cigarette smoking, 7% for current cigarette smoking, 20% for lifetime smoking of e-cigarettes, and 13% for current smoking of e-cigarettes. Independent positive correlates of both cigarette and e-cigarette smoking included male gender, age 15, and pertinence to more affluent families (all *p* < 0.01). The prevalence of smoking among Albanian children is seemingly high, which should be a cause of serious concern to decisionmakers and policymakers in this transitional country. Albania should immediately consider the implementation of expanded comprehensive tobacco control measures, which will save lives, reduce illness, and help reduce the economic burden associated with tobacco-related illness.

## 1. Introduction

Fairly recently, the World Health Organization (WHO) reiterated the fact that substance use, including smoking among children and adolescents, continues to be a significant public health concern due to its association with adverse health, behavioural, economic, and social consequences in the short, medium, and long term [1]. Negative consequences related to substance use among children consist of a wide range of psychological and physical health adversities, poorer academic performance, violence and injury, accidents, and detrimental effects on cognitive, emotional, and social development [1,2,3,4,5]. Furthermore, substance use (including smoking) among teenagers is often linked to other risk behaviours, resulting in the occurrence of multiple risky behaviours [6,7,8,9].

Within substance use, tobacco is a leading cause of preventable mortality and morbidity worldwide [10]. In 2019, about one billion people globally were regular tobacco users, with nearly eight million attributable deaths [10]. Tobacco use often begins during adolescence, with most adult smokers having started before the age of 21 [11]. According to the WHO, during the period 2010–2020, the average global prevalence of current use of any tobacco product among children aged 13–15 years was 10.3%, of which cigarette smoking comprised 6.0%, whereas smokeless tobacco constituted 2.6% [12]. Hence, while cigarette smoking constitutes the most prevalent tobacco product used by children and adolescents worldwide, there is an emerging growth and expansion of other tobacco products, particularly electronic cigarettes (e-cigarettes) [12,13]. Notably, the prevalence of tobacco use is relatively high among youth in low- and middle-income countries including Albania [11], a small post-communist country in the Western Balkans. In addition, there is evidence of a significant increase in tobacco product use among 13–15-year-old children in Albania during the period 2012–2020 [14]. Also, it has been reported that the use of e-cigarettes constitutes a growing concern in Albania, a situation that is similar to many other European countries [15].

The Health Behaviour in School-aged Children (HBSC) study is a large school-based survey conducted periodically (every four years) in many European countries and beyond since the mid-1980s [16,17,18]. The HBSC survey collects important information on several health behaviours, health outcomes, and the social environments of children aged 11, 13, and 15 years [16,17,18]. The last round of HBSC surveys was conducted in 2021–2022 in 44 countries including Albania [16,17]. In addition to a multitude of health outcomes and behavioural factors, the survey also contained a series of questions measuring smoking status among children aged 11, 13, and 15 years [18].

Albania, a post-communist country in the Western Balkans, has been described as a patriarchal society [19]. The country is currently in a continuous state of transition associated with enormous changes in the family structure and societal norms and values [19]. The available evidence indicates a high prevalence of child abuse in Albania, and lifetime smoking has been reported as an independent correlate of physical abuse among 15-year-old children [19]. The current legislation in Albania prohibits the selling of tobacco products to minors (<18 years) and indoor tobacco smoking (including e-cigarettes), but law enforcement falls short, especially outside Tirana (the capital).

In this context, considering the fact that the tobacco industry is actively targeting the underage market to sustain its profits, the objective of our study was to assess the prevalence and sociodemographic correlates of smoking among children in Albania, based on the data obtained from the last round of HBSC surveys conducted in 2022. In line with the evidence obtained from the previous rounds of this type of survey, we hypothesised a higher prevalence of smoking among boys and older children. 

## 2. Materials and Methods

The last round of HBSC surveys in Albania was conducted in 2022, consisting of a cross-sectional study employing an internationally standardised instrument [18], which was validated in Albania in 2009–2010 [19].

The study participants, drawn according to the HBSC international protocol [18], consisted of a nationwide representative sample (stratified multistage cluster sampling with probability proportional to size—the sampling frame was available from the Albanian Ministry of Education and Sport) of 5700 schoolchildren aged 11, 13, and 15 years (representing about 5% of the overall number of Albanian children belonging to this age-group). Of these, 246 children refused to participate in the survey and/or provided substantially incomplete or non-valid information. Hence, the study sample included 5454 schoolchildren aged 11, 13, and 15 years (2844 girls, or ≈52% of the overall sample). The overall response rate was 5454/5700 = 95.7%.

A structured self-administered paper questionnaire included, among other health behavioural characteristics, an assessment of smoking status, along with information on the sociodemographic characteristics of the schoolchildren.

The measurement of smoking status included the assessment of cigarette smoking (lifetime and in the last 30 days preceding the survey) and the assessment of the smoking of e-cigarettes (likewise, lifetime and in the last 30 days preceding the survey).

- *Lifetime cigarette smoking:* Schoolchildren were asked how many days (if any) they had smoked cigarettes in their lifetime. Potential responses were as follows: never, 1–2 days, 3–5 days, 6–9 days, 10–19 days, 20–29 days, and ≥30 days. In the analysis, lifetime cigarette smoking was dichotomised into “No” (never) vs. “Yes” (≥1–2 days/lifetime).
- *Cigarette smoking in the last 30 days preceding the survey:* Schoolchildren were asked how many days (if any) they had smoked cigarettes in the last 30 days preceding the survey. Potential responses were as follows: never, 1–2 days, 3–5 days, 6–9 days, 10–19 days, 20–29 days, and ≥30 days. In the analysis, this variable was dichotomised into “No” (never) vs. “Yes” (≥1–2 days/last 30 days). In this article, this variable is referred to as *“current cigarette smoking”*.
- *Lifetime smoking of e-cigarettes:* Schoolchildren were asked how many days (if any) they had smoked e-cigarettes in their lifetime. Potential responses were as follows: never, 1–2 days, 3–5 days, 6–9 days, 10–19 days, 20–29 days, and ≥30 days. In the analysis, lifetime smoking of e-cigarettes was dichotomised into “No” (never) vs. “Yes” (≥1–2 days/lifetime).
- *Smoking of e-cigarettes in the last 30 days preceding the survey:* Schoolchildren were asked how many days (if any) they had smoked e-cigarettes in the last 30 days preceding the survey. Potential responses were as follows: never, 1–2 days, 3–5 days, 6–9 days, 10–19 days, 20–29 days, and ≥30 days. In the analysis, this variable was dichotomised into “No” (never) vs. “Yes” (≥1–2 days/last 30 days). In this article, this variable is referred to as *“current smoking of e-cigarettes”*.

The sociodemographic characteristics (Table 1) consisted of children’s gender (boys vs. girls), age (11, 13, and 15 years), place of residence (urban vs. rural areas), mother’s and father’s current employment status (each dichotomised into yes vs. no), and family affluence scale [a composite index including family car ownership, children’s own bedroom, number of computers owned in the family, number of bathrooms at home, and the number of times children travelled with their families for a holiday in the past 12 months preceding the survey; the range of the index is from 6 (“least affluent families”) to 19 (“most affluent families”). In the analysis, the family affluence scale was dichotomised based on the median value of the summary score for each participant: “less affluent families” (below median, scores: 6–11) vs. “more affluent families” (above median, scores: 12–19)] [18].

The study was approved by the Ethics Committee of Tirana Medical University (approval ID: No. 700/1) and the data collection process was also confirmed by the Albanian Ministry of Education and Sport. All schoolchildren were informed about the aim and objectives of the study and were explained in sufficient detail, particularly the aspects related to the anonymity of the survey and the successive aggregated data analysis. Passive consent was sought from the parents through teachers from each respective school.

Binary logistic regression was used to assess the association of smoking variables with the sociodemographic characteristics of the schoolchildren (Table 2 and Table 3). Initially, crude (unadjusted) models were run (Table 2). Odds ratios (ORs) and their respective 95% confidence intervals (95%CIs) and *p*-values (P) were calculated for each smoking variable. Next, all sociodemographic factors (gender, age, residence, mother’s and father’s employment, and family affluence scale) were entered simultaneously into the logistic regression models (Table 3). Multivariable-adjusted ORs and their respective 95%CIs and *p*-values were calculated for each of the four smoking variables (lifetime cigarette smoking, current cigarette smoking, lifetime smoking of e-cigarettes, and current smoking of e-cigarettes). For all multivariable-adjusted logistic regression models, a Hosmer–Lemeshow test was used to assess the goodness-of-fit; all models fit the criterion [20]. 

For all statistical tests employed, *p* ≤ 0.05 was considered statistically significant. Statistical Package for the Social Sciences (SPSS, version 19.0, IBM Corp, Armonk, NY, USA) was used for all statistical analyses.

## 3. Results

Table 1 presents the distribution of smoking variables according to the sociodemographic characteristics of Albanian schoolchildren included in the HBSC 2022 survey. About one in three schoolchildren were aged 11, 13, or 15 years, respectively; almost one in three participants resided in rural areas; one in three children reported that their mothers were unemployed, whereas their fathers’ unemployment was reported by almost one in ten of the children; and almost half of schoolchildren were from less affluent families.

**Table 1:** Distribution of smoking variables by sociodemographic characteristics in a nationwide sample of Albanian schoolchildren, HBSC 2022 survey.

Sociodemographic Characteristics	Lifetime Cigarette Smoking (≥1–2 Days/Lifetime)	Current Cigarette Smoking (≥1–2 Days/Last 30 Days)	Lifetime Smoking of e-Cigarettes (≥1–2 Days/Lifetime)	Current Smoking of e-Cigarettes (≥1–2 Days/Last 30 Days)
Total sample (n = 5454)	646 (12.1) ^1^	380 (7.1)	1020 (19.5)	690 (13.0)
Gender:				
Boys (n = 2610)	386 (15.2)	242 (9.4)	649 (26.2)	444 (17.6)
Girls (n = 2844)	260 (9.3)	138 (4.9)	371 (13.4)	246 (8.8)
Age:				
11 years (n = 1784)	76 (4.3)	43 (2.4)	134 (7.8)	81 (4.7)
13 years (n = 1785)	180 (10.3)	107 (6.1)	315 (18.4)	217 (12.5)
15 years (n = 1877)	387 (21.2)	229 (12.4)	570 (31.6)	391 (21.4)
Residence:				
Urban areas (n = 3648)	451 (12.6)	259 (7.2)	687 (19.6)	470 (13.2)
Rural areas (n = 1806)	195 (11.1)	121 (6.8)	333 (19.1)	220 (12.6)
Father’s employment:				
Yes (n = 4928)	580 (12.1)	345 (7.1)	927 (19.6)	621 (13.0)
No (n = 479)	61 (13.0)	31 (6.5)	88 (18.9)	65 (13.8)
Mother’s employment:				
Yes (n = 3676)	448 (12.5)	262 (7.2)	720 (20.3)	492 (13.7)
No (n = 1710)	189 (11.3)	111 (6.6)	287 (17.6)	189 (11.5)
Family affluence:				
Less affluent (n = 2600)	263 (10.3)	134 (5.2)	397 (15.8)	254 (10.0)
More affluent (n = 2715)	360 (13.6)	232 (8.6)	597 (23.0)	418 (15.8)

^1^ Absolute numbers and their respective percentages (in parentheses). For smoking variables, there were the following missing values: lifetime cigarette smoking (n = 128), current cigarette smoking (n = 73), lifetime smoking of e-cigarettes (n = 213), and current smoking of e-cigarettes (n = 147). Conversely, for sociodemographic factors, there were the following missing values: age of schoolchildren (n = 8), father’s employment status (n = 47); mother’s employment status (n = 68); and family affluence score (n = 139).

Overall, the prevalence of lifetime cigarette smoking was about 12% (Table 1). It was higher in boys than in girls (around 15% vs. 9%, respectively) and among children from more affluent families than in those from poorer families (about 14% vs. 10%, respectively). There were no significant differences regarding place of residence or parental employment status, but a graded positive relationship with age, with older children exhibiting a much higher prevalence of lifetime cigarette smoking (21%) compared to their younger counterparts (only about 4%). The overall prevalence of current cigarette smoking was about 7%, exhibiting a similar pattern of sociodemographic distribution as the lifetime cigarette smoking, with boys, children from more affluent families, and especially older children displaying higher levels of current smoking. On the whole, the prevalence of lifetime smoking of e-cigarettes was about 20%. It was higher in boys than in girls (about 26% vs. 13%, respectively), in children from more affluent families than in those from poorer families (23% vs. 16%, respectively) and, in particular, among older children compared to their younger counterparts (32% vs. 8%, respectively). The overall prevalence of current smoking of e-cigarettes was 13%, with a similar sociodemographic distribution as the lifetime index (Table 1).

In the crude/unadjusted binary logistic regression models (Table 2), lifetime cigarette smoking was positively associated with male gender (OR = 1.7, 95%CI = 1.5–2.1) and especially older age of schoolchildren (OR = 5.9, 95%CI = 4.6–7.7) [upper panel]. Conversely, there was an inverse relationship with belonging to less wealthy families (OR = 0.7, 95%CI = 0.6–0.9) [lower panel]. A very similar pattern was evident for current smoking (Table 2). Lifetime smoking of e-cigarettes was positively associated with male gender (OR = 2.3, 95%CI = 2.0–2.6) and especially with older age (OR = 5.5, 95%CI = 4.5–6.7) [upper panel], but inversely related to maternal unemployment (OR = 0.8, 95%CI = 0.7–1.0) and belonging to less wealthy families (OR = 0.6, 95%CI = 0.5–0.7) [lower panel]. Almost identical estimates were evident for current smoking of e-cigarettes (Table 2).

**Table 2:** Association of smoking variables with sociodemographic characteristics of schoolchildren; results from *unadjusted* binary logistic regression models.

**Upper Panel: Demographic Factors**						
**Smoking Variables**	**Demographic Variables**					
	**Male**		**Age 15**		**Urban Areas**	
	**OR** **(95%CI) ^1^**	***p* ^1^**	**OR** **(95%CI)**	***p***	**OR** **(95%CI)**	***p***
Lifetime cigarette smoking (≥1–2 days/lifetime)	1.75 (1.48–2.07)	<0.001	5.94 (4.60–7.67)	<0.001	1.16 (0.97–1.39)	0.099
Current cigarette smoking (≥1–2 days/last 30 days)	2.02 (1.63–2.51)	<0.001	5.64 (4.04–7.86)	<0.001	1.07 (0.85–1.33)	0.573
Lifetime smoking of e-cigarettes (≥1–2 days/lifetime)	2.29 (1.98–2.63)	<0.001	5.48 (4.47–6.70)	<0.001	1.03 (0.89–1.20)	0.666
Current smoking of e-cigarettes (≥1–2 days/last 30 days)	2.21 (1.87–2.61)	<0.001	5.55 (4.32–7.12)	<0.001	1.06 (0.89–1.26)	0.499
**Lower Panel: Socioeconomic Characteristics**						
**Smoking Variables**	**Socioeconomic Variables**					
	**Father Unemployed**		**Mother Unemployed**		**Less Affluent**	
	**OR** **(95%CI) ^1^**	***p* ^1^**	**OR** **(95%CI)**	***p***	**OR** **(95%CI)**	***p***
Lifetime cigarette smoking (≥1–2 days/lifetime)	1.09 (0.82–1.45)	0.546	0.89 (0.75–1.07)	0.221	0.73 (0.61–0.86)	<0.001
Current cigarette smoking (≥1–2 days/last 30 days)	0.91 (0.62–1.33)	0.625	0.90 (0.72–1.13)	0.372	0.58 (0.47–0.73)	<0.001
Lifetime smoking of e-cigarettes (≥1–2 days/lifetime)	0.96 (0.75–1.22)	0.730	0.84 (0.72–0.97)	0.021	0.63 (0.55–0.72)	<0.001
Current smoking of e-cigarettes (≥1–2 days/last 30 days)	1.08 (0.82–1.42)	0.592	0.82 (0.68–0.98)	0.026	0.59 (0.50–0.70)	<0.001

^1^ Odds ratios and their respective 95% confidence intervals (in parentheses), as well as *p*-values from *crude (unadjusted)* binary logistic regression models. Reference categories (groups) were as follows: “female” (for gender), “age 11” (for age), “rural areas” (for place of residence), “father employed” (for paternal employment), “mother employed” (for maternal employment), and “more affluent” (for family affluence scale).

In multivariable-adjusted binary logistic regression models (Table 3), independent and significant correlates of lifetime cigarette smoking consisted of male gender (OR = 1.9, 95%CI = 1.6–2.3), age 15 (OR = 5.9, 95%CI = 4.6–7.7) [upper panel], and belonging to less affluent families (OR = 0.8, 95%CI = 0.7–0.9) [lower panel]. Similarly, independent and significant correlates of current cigarette smoking included male gender (OR = 2.2, 95%CI = 1.7–2.7), age 15 (OR = 5.6, 95%CI = 4.0–7.9) [upper panel], and belonging to less affluent families (OR = 0.7, 95%CI = 0.5–0.8) [lower panel]. Furthermore, independent correlates of lifetime smoking of e-cigarettes consisted of male gender (OR = 2.5, 95%CI = 2.2–2.9), older age (OR = 5.9, 95%CI = 4.8–7.3) [upper panel], and belonging to less wealthy families (OR = 0.7, 95%CI = 0.6–0.8) [lower panel]. Likewise, independent and significant correlates of current smoking of e-cigarettes included male gender (OR = 2.4, 95%CI = 2.0–2.8), age 15 (OR = 5.8, 95%CI = 4.4–7.4) [upper panel], and belonging to less affluent families (OR = 0.6, 95%CI = 0.5–0.8) [lower panel].

**Table 3:** Independent sociodemographic correlates of smoking variables; results from *multivariable-adjusted* binary logistic regression models.

**Upper Panel: Demographic Factors**						
**Smoking Variables**	**Demographic Variables**					
	**Male**		**Age 15**		**Urban Areas**	
	**OR** **(95%CI) ^1^**	***p* ^1^**	**OR** **(95%CI)**	***p***	**OR** **(95%CI)**	***p***
Lifetime cigarette smoking (≥1–2 days/lifetime)	1.88 (1.58–2.25)	<0.001	5.92 (4.55–7.71)	<0.001	1.07 (0.89–1.30)	0.467
Current cigarette smoking (≥1–2 days/last 30 days)	2.18 (1.73–2.73)	<0.001	5.57 (3.95–7.85)	<0.001	0.95 (0.75–1.20)	0.659
Lifetime smoking of e-cigarettes (≥1–2 days/lifetime)	2.52 (2.16–2.93)	<0.001	5.91 (4.78–7.30)	<0.001	0.91 (0.78–1.07)	0.248
Current smoking of e-cigarettes (≥1–2 days/last 30 days)	2.37 (1.99–2.82)	<0.001	5.75 (4.44–7.44)	<0.001	0.95 (0.79–1.14)	0.584
**Lower Panel: Socioeconomic Characteristics**						
**Smoking Variables**	**Socioeconomic Variables**					
	**Father Unemployed**		**Mother Unemployed**		**Less Affluent**	
	**OR** **(95%CI) ^1^**	***p* ^1^**	**OR** **(95%CI)**	***p***	**OR** **(95%CI)**	***p***
Lifetime cigarette smoking (≥1–2 days/lifetime)	1.11 (0.82–1.51)	0.489	0.93 (0.76–1.12)	0.428	0.78 (0.65–0.93)	0.006
Current cigarette smoking (≥1–2 days/last 30 days)	1.02 (0.68–1.52)	0.941	0.97 (0.76–1.23)	0.771	0.65 (0.51–0.81)	<0.001
Lifetime smoking of e-cigarettes (≥1–2 days/lifetime)	1.05 (0.81–1.37)	0.714	0.87 (0.74–1.02)	0.094	0.67 (0.58–0.78)	<0.001
Current smoking of e-cigarettes (≥1–2 days/last 30 days)	1.25 (0.93–1.68)	0.137	0.85 (0.70–1.03)	0.090	0.64 (0.54–0.77)	<0.001

^1^ Odds ratios and their respective 95% confidence intervals (in parentheses), as well as *p*-values from *multivariable-adjusted* binary logistic regression models. All sociodemographic factors (gender, age, residence, mother’s and father’s employment, and family affluence scale) were entered simultaneously into the models. Reference categories (groups) were as follows: “female” (for gender), “age 11” (for age), “rural areas” (for place of residence), “father employed” (for paternal employment), “mother employed” (for maternal employment), and “more affluent” (for family affluence scale).

A separate analysis by gender provided essentially the same evidence; i.e., independent positive correlates of cigarette and e-cigarette smoking in both genders consisted of age 15 and belonging to more affluent families.

## 4. Discussion

The main findings of this study consist of a strong and independent link between smoking status and several sociodemographic characteristics of Albanian schoolchildren. Hence, male gender, age 15, and belonging to a more affluent family were each strongly and positively associated with both cigarette and e-cigarette smoking irrespective of other sociodemographic factors. On the whole, the prevalence of smoking variables was 12% for lifetime cigarette smoking, 7% for current cigarette smoking, 20% for lifetime smoking of e-cigarettes, and 13% for current smoking of e-cigarettes. Overall, the prevalence of smoking among Albanian children is apparently high, and this is a cause for concern when also taking into account that the tobacco industry deliberately aims to attract juvenile consumers to maintain its profit margins. 

Our findings on the prevalence of lifetime cigarette smoking in boys and 11-year-old girls (not shown in the tables) are similar to the recent WHO multi-country report on the findings of the last HBSC round conducted in 44 countries in 2021–2022 [1]. Hence, the prevalence of lifetime cigarette smoking among 11-year-old boys in our study was 6% vs. 5% in the multi-country report [1]; among 13-year-old boys in our study was 13% vs. 10% in the multi-country report [1]; among 15-year-old boys in our study was 26% vs. 24% in the multi-country report [1]; and among 11-year girls in our study was 3%, same as the average of the multi-country report [1]. On the other hand, our findings on the prevalence of lifetime smoking in girls aged 13 years, and particularly those aged 15 years, are lower than the multi-country average: 7% vs. 11%, respectively, and 17% vs. 26%, respectively [1]. 

Regarding time trends, on average, lifetime cigarette smoking declined between 2018 and 2022 (last HBSC round), especially among 13-year-old boys and 15-year-old boys and girls [1]. However, this is not the case for Albania, which exhibits an almost identical prevalence in the last three HBSC rounds conducted in 2014, 2018, and 2022. Furthermore, the prevalence of current cigarette smoking slightly increased among Albanian schoolchildren in 2022 compared with the previous HBSC rounds.

In our study, there was evidence of a positive relationship between family affluence and smoking, somehow contrary to most countries and regions that, in the last HBSC round conducted in 2021–2202, displayed no significant differences in lifetime and current smoking according to the socioeconomic status of the schoolchildren [1]. We believe that our findings of a positive link between family affluence and smoking relate to the greater purchasing power of children from more affluent (wealthy) families, especially for e-cigarettes, which are more costly. As a matter of fact, our argument is supported by the significantly higher prevalence of smoking of e-cigarettes among children from more affluent families in ten countries included in the last HBSC round [1]. Furthermore, several previous studies conducted in many countries have reported a higher prevalence of smoking among adolescents receiving a higher amount of pocket money [15,21,22,23]. Hence, it has been suggested that having disposable income may lead to easier access to tobacco products, thereby increasing the levels of tobacco use [15,21,24]. Also, a positive relationship has been reported between smoking in children and the lower price of a pack of cigarettes [21].

Regarding the smoking of e-cigarettes, the lifetime prevalence in the multi-country report [1] was 18%, which is somewhat similar to our study conducted in Albania (20%), whereas the prevalence of current smoking was 10% (compared with 13% in our study). Of note, a measurement of e-cigarette smoking among Albanian children was included for the first time in the HBSC 2022 round. At an international level, while the available evidence indicates that tobacco use among youth remained unchanged between 2012 and 2020, the smoking of e-cigarettes increased in many countries [14].

In our study, similar to the multi-country report [1], the prevalence of smoking (both for cigarettes and e-cigarettes) increased significantly with the age of the schoolchildren. Indeed, this is the case for almost all countries and regions of the world [1].

In our study, boys had a significantly higher prevalence of smoking (for both cigarettes and e-cigarettes) compared to girls, contrary to most of the other countries where no significant difference between boys and girls was found, particularly for current smoking [1]. The higher smoking rates among boys compared to girls in our study may be explained by the cultural norms and gender roles in Albania, including masculinity and social expectations, as well as the gender-specific socialisation (boys in Albania are exposed to environments where smoking is “normalised”, whereas girls are more socially isolated). Also, another factor may be related to the underreporting of smoking among Albanian girls due to the stigma related to this behaviour in patriarchal societies such as Albania. 

On another note, dual- and poly-use of tobacco products are emerging behaviours among children and adolescents worldwide [25,26]. As an illustration, thee current smoking of cigarettes and e-cigarettes has been reported as the most prevalent tobacco use behaviour among adolescents in Ireland [25]. This trend will unavoidably affect Albanian youth too and, therefore, there is a need to closely monitor the emerging trends of new tobacco products in order to ensure progress toward the effective control and prevention of tobacco use. 

Our study included a large nationwide representative sample of schoolchildren (with a very high response rate) and was based on a standardised international instrument previously validated in Albania [19]. Nevertheless, a potential limitation of our study may consist of the lack of generalisability to out-of-school children of this age group (11, 13, and 15 years) in Albania. Additionally, children aged 12 or 14 years were not included in the survey, as based on the HBSC protocol; only three age groups of children are chosen according to time periods that represent the following: the onset of adolescence (age 11); the challenge of physical and emotional change (age 13); and the years when very important life and career decisions are beginning to be made (age 15) [16,17,18]. Also, the possibility of information bias should be considered, including social desirability bias (underreporting of smoking by the children due to stigma), recall bias (particularly for lifetime smoking, as children may not accurately remember infrequent smoking behaviour occurring a long time ago), or misreporting (the intentional provision of false information). Also, the cross-sectional study design may pose another limitation as it does not allow for drawing causal conclusions.

Nonetheless, our study provides valuable evidence on the current status of smoking among schoolchildren in Albania, a country that is undergoing a profound transformation associated with changes in behavioural patterns of the general population including children [27]. Policymakers should use the data generated from national surveys to strengthen existing tobacco and e-cigarette legislation and public policies in Albania and to promote a substantial increase in tobacco taxes. This is the most cost-effective strategy to prevent the uptake of tobacco and e-cigarettes among young people, especially those with lower incomes. Also, our findings highlight the importance of regular monitoring to better understand the public health impact of tobacco use among children and adolescents in Albania, considering that the tobacco industry is actively seeking to capture the underage market to continue its profits, despite the adverse health and economic consequences that this global epidemic causes in low- and middle-income countries. Indeed, the monitoring of smoking trends and other behavioural characteristics is important at an international scale, as a fairly recent report indicates that over 60% of students exhibit two or more modifiable risk factors for non-communicable diseases, including smoking [28]. 

## 5. Conclusions

The prevalence of lifetime cigarette smoking among the Albanian schoolchildren included in this study was 12%, whereas the prevalence of the lifetime smoking of e-cigarettes was 20%. Independent positive correlates of both cigarette and e-cigarette smoking included male gender, age 15, and belonging to more affluent families. Hence, our study indicates that the prevalence of smoking among Albanian children is seemingly high, which should be a cause of serious concern for decisionmakers and policymakers in this transitional country. The tobacco epidemic among Albanian schoolchildren bears short-and long-term consequences on the burden of disease and significant economic consequences for the health system. At an international level, our findings advocate for the need to strengthen global monitoring and surveillance strategies for the tobacco epidemic and the introduction of e-cigarettes that affect the most vulnerable population groups, including adolescents.

In conclusion, Albania should immediately consider the implementation of expanded comprehensive tobacco control measures, which will save lives, reduce illness, and help reduce the economic burden associated with tobacco-related illness and lost productivity. Also, stakeholders are encouraged to implement multi-level and multi-modal school-based programmes to prevent the initiation of tobacco and e-cigarette use among Albanian youth, which can be applied synergistically with other preventable risk factors. 

## Data Availability

The data presented in this study are available on request from the corresponding author.
